# Aluminum Hydroxide Adjuvant Differentially Activates the Three Complement Pathways with Major Involvement of the Alternative Pathway

**DOI:** 10.1371/journal.pone.0074445

**Published:** 2013-09-09

**Authors:** Esin Güven, Karen Duus, Inga Laursen, Peter Højrup, Gunnar Houen

**Affiliations:** 1 Department of Clinical Biochemistry, Immunology and Genetics, Statens Serum Institut, Copenhagen, Denmark; 2 Department of Biochemistry and Molecular Biology, University of Southern Denmark, Odense, Denmark; University of Kentucky College of Medicine, United States of America

## Abstract

Al(OH)_3_ is the most common adjuvant in human vaccines, but its mode of action remains poorly understood. Complement involvement in the adjuvant properties of Al(OH)_3_ has been suggested in several reports together with a depot effect. It is here confirmed that Al(OH)_3_ treatment of serum depletes complement components and activates the complement system. We show that complement activation by Al(OH)_3_ involves the three major pathways by monitoring complement components in Al(OH)_3_-treated serum and in Al(OH)_3_-containing precipitates. Al(OH)_3_ activation of complement results in deposition of C3 cleavage products and membrane attack complex (MAC) and in generation of the anaphylatoxins C3a and C5a. Complement activation was time dependent and inhibited by chelation with EDTA but not EGTA+Mg^2+^. We thus confirm that Al(OH)_3_ activates the complement system and show that the alternative pathway is of major importance.

## Introduction

Immunological adjuvants are compounds which initiate and boost immune responses, leading to stronger and faster adaptive immune responses without having any antigenic effect by themselves [1], [2]. Many different compounds of both organic and inorganic origin have been observed to stimulate a vigorous immune response and therefore have adjuvant properties; these include mineral oils and different metal salts, notably aluminum compounds (e.g. aluminium hydroxide (Al(OH)_3_), the hydrated form of aluminium oxide (Al_2_O_3_) [2]–[6]. Also, the pathogen-associated molecular patterns (PAMPs) are a big group of naturally occurring compounds with adjuvant properties. They include CpG DNA, ssDNA, dsRNA and bacterial cell wall components [7], [8].

The first adjuvant effect of an aluminum compound was described by Glenny et al. [9] and the effect has been used in vaccines since the first half of the 20^th^ century (from around 1930). Among the few approved adjuvants for human vaccines the aluminum compounds are often preferred and have been used extensively for diphtheria vaccines, tetanus vaccines, pertussis vaccines, hepatitis vaccines and polio vaccines [10]–[12].

**Figure 1:**
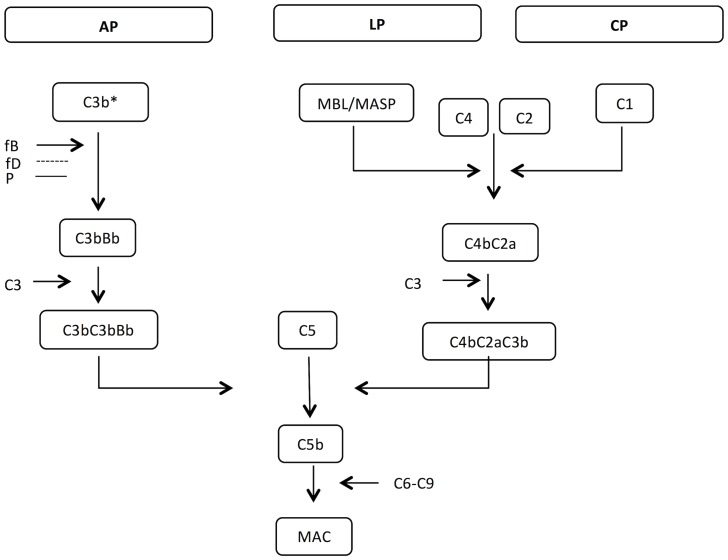
The three pathways of complement activation. The alternative pathway (AP) is activated when C3 undergoes spontaneous hydrolysis and forms the initial C3 convertase, C3(H_2_O)Bb in the presence of factor B (fB) and cleavage of bound fB by factor D (fD). The alternative C3-convertase is stabilized by properdin (P). The C3 convertase generates C3b and the subsequent C3-convertases are assembled by C3b and Bb. The lectin pathway (LP) is activated when MBL or other immune lectins bind carbohydrates on pathogens, activating the associated serine proteases (MASPs) which cleave C4 and C2. The first component of the classical pathway (CP) is C1, a complex of C1q and its associated serine proteases C1r and C1s. The CP is initiated by C1q recognition of immune complexes, activating C1r and C1s, again cleaving C4 and C2 generating a C3 convertase characteristic of the CP and the LP (C4bC2a). The C3 convertase cleaves C3 generating C3b and enables assembly of a C5 convertase (C3bC3bBb or C4bC2aC3b), and the C5-convertase product (C5b) initiates assembly of the membrane attack complex (MAC) from C5b-C9. * Initial alternative C3 convertase generated from C3(H_2_O) and fB.

The modes of action of aluminum adjuvants are still a subject of research but several mechanisms have been suggested. Glenny et al. initially described the adjuvant effect of aluminium adjuvants to be due to the ability of these to form a depot and to control the release of antigen [13]; this has later been questioned [14]. Other non-exclusive modes of action have also been suggested, including enhancement of antigen uptake and presentation, innate immune system activation and enhancement of cytokine production and release [1]–[4], [15]–[18]. Recently, aluminum hydroxide has been observed to bind lipid moieties on dendritic cells and promote lipid sorting in the plasma membrane, leading to signal transduction and immune response initiation [19] and increase antigen uptake and enhance antigen presentation on dendritic cells [20], [21] and directly affect B lymphocytes [22]. Aluminum hydroxide has also been described to be able to activate the complement system. Already in 1975, Polley and Nachman observed that aluminum hydroxide could remove 40–60% of the haemolytic complement activity in a serum sample [23]. This was later confirmed by Ramanathan et al., who found that aluminum and zirconium compounds could activate the complement system and it was suggested that the complement activation occurred through the lectin pathway [24]. However, findings by Arvidsson et al., suggested that an aluminum surface binds C3 through the classical complement pathway [25]. On the contrary, Tengvall et al., found no evidence that complement deposition on aluminium hydroxide occurred as a result of complement activation [26] (the three major complement pathways are described in Fig. 1).

Here, we confirm that Al(OH)_3_ activates the complement system and show that aluminum hydroxide adjuvant activates the three complement pathways with major involvement of the alternative complement pathway, thus providing a rationale for its efficient adjuvant properties.

## Materials and Methods

### Chemicals and Proteins

Al(OH)_3_ (Alhydrogel®, sterile and free of pyrogens) was from Brenntag Biosector (Frederikssund, Denmark). TTN-buffer (0.05 M Tris, 1% Tween 20, 0.3 M NaCl, pH 7.5), alkaline phosphatase substrate buffer, human serum albumin (HSA), DiTe booster vaccine (difteria-tetanus toxoid vaccine), monoclonal antibodies against factor H, properdin, factor B, mannan-binding lectin (MBL), C5, C4, C3d and beta galactosidase were prepared in-house at SSI (Copenhagen, Denmark). Monoclonal antibody against C1q was from Quidel (San Diego, CA, USA) and monoclonal antibody against L ficolin was from BioPorto diagnostics A/S (Gentofte, Denmark). Bovine serum albumin (BSA), alkaline phosphatase-conjugated secondary antibodies (goat anti-mouse IgG), alpha-cyano-4-hydroxycinnamic acid, BCIP/NBT (5-bromo-4-chloro-3-indolyl phosphate/nitroblue tetrazolium chloride) tablets and alkaline phosphatase substrate tablets were from Sigma-Aldrich (St. Louis, Missouri, USA). Mouse anti-human C5b-C9 (membrane attack complex (MAC)) and rabbit anti-human C3c were from Dako (Glostrup, Denmark). Trypsin was from Promega (Madison, USA) and iC3b was from EMD Chemicals (San Diego, CA, USA).

### Sera

Normal human serum (NHS) was collected from healthy volunteers and used anonymously and pooled. The serum pool was found to be fully competent in all three complement pathways (Table 1), Heat-inactivated NHS (HIS) was prepared by incubation at 56°C for 30 minutes. MBL-deficient serum was from a donor found to be completely restricted in the lectin pathway (Table 1). The sera were from the Biomarker and Immunology (BMI) Biobank at Statens Serum Institut and have been donated anonymously for research purposes.

**Table 1:** MAC deposition of human complement-competent and -compromised sera in the three individual complement pathways.

Serum (source)	Pathway (cut-off)		
	CP (>69)	LP (>10)	AP (>30)
Healthy control			
serum (NHS)	91±6	80±7	96±31
(In-house)			
Heat-inactivated			
serum (HIS)	0±0	0±0	0±0
(In-house)			
C3-depleted			
serum	2±1	1±1	0±0
(Sigma)			
MBL-deficient			
serum	69±6	0±0	31±12
(In-house)			
Factor B-depleted			
serum	58±6	88±12	0±0
(Sigma)			

Values are represented as means±SD of 3 independent experiments. Normal complement function is above 69, 10 and 30 units for the CP, LP and AP respectively, as indicated in the table. MAC, membrane attack complex; NHS, normal healthy control serum; HIS, heat inactivated serum; MBL, mannan binding lectin; CP, classical pathway; LP, lectin pathway; AP, alternative pathway.

C3-depleted serum and factor B-depleted serum were from Sigma-Aldrich (St. Louis, Missouri, USA).

Goat serum was from SSI Diagnostica (Hillerød, Denmark).

### Complement Activity Assays

The activities of the three major complement pathways (Figure 1, Table 1) were determined using a commercial assay from Euro-diagnostica (Malmö, Sweden), following the manufacturer’s instructions. This assay has been described previously [27], [28]. In brief, serum was diluted (AP 1∶18, LP and CP 1∶101) in pathway specific buffers (AP: 1.8 mM Na-5,5-diethylbarbital, 0.2 mM 5,5-diethylbarbituric acid, 145 mM NaCl, 10 mM EGTA, 5 mM MgCl_2_, 0.05% Tween-20, 0.1% gelatin, LP: 1.8 mM Na-5,5-diethylbarbital, 0.2 mM 5,5-diethylbarbituric acid, 145 mM NaCl, 0.5 mM MgCl_2_, 2 mM CaCl_2_, 0.05% Tween-20, 0.1% gelatin, 20 µg/ml mab 2204 (inhibitor of CP), CP: 1.8 mM Na-5,5-diethylbarbital, 0.2 mM 5,5-diethylbarbituric acid, 145 mM NaCl, 0.5 mM MgCl_2_, 2 mM CaCl_2_, 0.05% Tween-20, 0.1% gelatin) and loaded in pre-coated wells of an ELISA plate together with a blank control, a positive and a negative control. After incubation the complement end product was quantified and complement activity calculated from included controls.

### Aluminum Hydroxide Experiments

Samples were mixed with Alhydrogel (2% (Al_2_O_3_)) in varying amounts and incubated as indicated at 37°C. The fluid phase and the amorphous Al(OH)_3_ precipitate were then separated by centrifugation and used for further analyses.

Alhydrogel is a trade name for a suspension of Al_2_O_3_ (2%) in water. When suspended in water, Al_2_O_3_ reacts with water (it is hydrated, so to speak) to form aluminum hydroxide, Al(OH)_3_, which has the form of an amorphous precipitate. Aluminum hydroxide is thus the name for the chemical entity Al(OH)_3_, i.e. the hydrated form of aluminum oxide. Aluminum hydroxide cannot be isolated as a solid compound, it only exists as the amorphous form. The concentration/amount of Al(OH)_3_ in a 2% (w:w) suspension of Al_2_O_3_ in water is 3% (w:w) corresponding to a concentration of 6 mM. There are no other constituents in Alhydrogel and the product is certified pyrogen free.

### Enzyme-Linked Immunosorbent Assay (ELISA)

Amorphous Al(OH)_3_ precipitate was resuspended in 100 µL MilliQ water and used for coating of microtitre plates 1∶10 in coating buffer (0.05 M sodium carbonate, pH 9.6) overnight at 2–6°C. Blocking of residual binding sites and washing was performed using TTN buffer (50 mM Tris, 1% Tween 20, 0.3 M NaCl, pH 7.5). Antibodies against complement components were diluted in TTN and added in duplicates at 1 µg/mL and incubated for 1 h at room temperature. Following 3× washing with TTN, the plate was incubated with alkaline phosphatase-conjugated secondary antibody diluted 1000-fold in TTN and incubated for 1 h. After another washing, bound antibodies were quantified by addition of *p*-nitrophenyl phosphate (1 mg/mL dissolved in 1 M diethanolamine, 0.5 mM MgCl_2_, pH 9.8). The colour development was measured after incubation at room temperature for 30 min at 405 nm with 650 nm as reference. Data are presented as mean values with error bars indicating the standard deviation.

### Sodium Dodecyl Sulphate-Polyacrylamide Gel Electrophoresis (SDS-PAGE)

SDS-PAGE was performed using precast 4–20% Tris-glycine gels according to the manufacturer’s instructions (Novex, Invitrogen, Tåstrup, Denmark). Resuspended amorphous Al(OH)_3_ precipitate was solubilised in 0.5 M EDTA and subjected to intensive dialysis in 0.05 M carbonate buffer with 10 mM EDTA, pH 9.6 to remove aluminum. Finally, it was mixed with an equal volume of sample buffer (0.05% Tris-HCl, 10% glycerol, 2% SDS, 0.1 M DTT. 0.0625% Pyronin G) and heated for 2 min at 85°C. After centrifugation, samples were loaded in the wells of the gel. Electrophoresis was performed for about 90 min at 125 V using Tris-glycine running buffer (25 mM Tris, 192 mM glycine, 0.1% SDS, pH 8.3). The gels were stained with GelCode Blue stain reagent (Thermo Scientific, Rockford, USA) according to the manufacturer’s recommendations.

### Immunoblotting

After electrophoresis, gels were blotted to 0.2 µm nitrocellulose membrane (Sigma-Aldrich, St. Louis, Missouri, USA) using Tris-glycine running buffer. Blotting was performed for 1 h at 200 mA and subsequently overnight at 20 mA. Membranes were blocked with TTN buffer containing 1% goat serum, and incubated for 1 h at room temperature with monoclonal antibody directed against C3d diluted 2000-fold in TTN buffer with 1% goat serum. After incubation, the membranes were washed and incubated with alkaline phosphatase-conjugated secondary antibody (goat anti mouse IgG) diluted 1000-fold in TTN buffer with 1% goat serum, for 1 h at room temperature. The strips were washed and incubated with BCIP/NBT substrate solution until appearance of bands.

### Mass Spectrometry

Coomassie Brilliant Blue-stained protein bands were excised and cut into 1 mm^3^ pieces, washed in 50% v/v acetonitrile (ACN), shrunk by dehydration in ACN, rehydrated in 0.1 M ammonium bicarbonate, pH 7.8, and finally shrunk again in ACN. The proteins were reduced with 10 mM DTT in 0.1 M ammonium bicarbonate for 30 min at 56°C after which alkylation was performed with 50 mM iodacetamide in 0.1 M ammonium bicarbonate for 30 min in the dark. The gel pieces were washed and dehydrated once more before addition of sequencing grade trypsin (12.5 ng/µL in 50 mM ammonium bicarbonate). After 10 min, surplus trypsin was removed and 20 µL of 50 mM ammonium bicarbonate was added to keep the gel pieces wet during the tryptic digestion performed at 50°C for one hour. Prior to analysis, peptide mixtures were desalted and concentrated on GELoader tip columns packed with Poros R2-50 reversed phase packing resin (PerSeptive Biosystems, Framingham, MA, USA). Bound peptides were eluted directly onto a MALDI target using 0.5 µL of 5 µg/µL alpha-cyano-4-hydroxycinnamic acid (HCCA) in 70% ACN, 0.1% trifluoroacetic acid (TFA).

The peptide mixture was analyzed by MALDI-TOF MS/MS on an ABI 4800 plus TOF-TOF instrument (Applied Biosystems, CA). External calibration was performed using a tryptic peptide mixture derived from bovine beta-lactoglobulin. Mass spectra were analysed by the software package MoverZ (m/z) (Genomic Solutions, Ann Arbor, MI, USA) and internally calibrated using the PeakErazor program (Lighthouse data, Odense, Denmark). Peak lists were searched against the Mascot NCBI database (http://matrix-science.com) using the PMF tool or the MS/MS ion search tool. Fixed modifications were set to carbamidomethylation of cysteines, and variable modifications to oxidation of methionine. Peptide mass tolerance was set to ±50 ppm, maximum missed cleavages by trypsin to 1, and all entries were included in the search.

### Quantification of C3, C4 and Immunoglobulins

The concentration of the immunoglobulins and C3 and C4 were measured by rate nephelometry on an Immage instrument (Beckman Coulter, Fullerton, USA) following the instructions of the manufacturer.

### Quantification of Anaphylatoxins

C3a, C4a, C5a were quantified by ELISA using commercial kits and following the instructions of the manufacturer (Quidel, San Diego, CA, USA).

### Statistics

All measurements were done as double determinations and experiments were repeated at least 2 times. Figures show means with error bars (standard deviations (SD)) of representative experiments. 2-way ANOVA and Bonferroni posttest were calculated on the means of duplicates of two-three independent experiments using Graphpad Prism (La Jolla, CA, USA).

## Results

### Al(OH)_3_ Exhausts Serum Complement

The activities of the three major complement pathways (alternative pathway (AP), lectin pathway (LP), classical pathway (CP)) (Fig. 1) were determined in NHS, HIS (NHS heated to 56°C for 30 min) and in three complement-compromised sera (Table 1). As expected, the MBL-deficient serum showed no LP activity and the factor B-depleted serum showed no AP activity. A C3-depleted serum showed no activity in any of the three pathways and neither did HIS.

**Figure 2:**
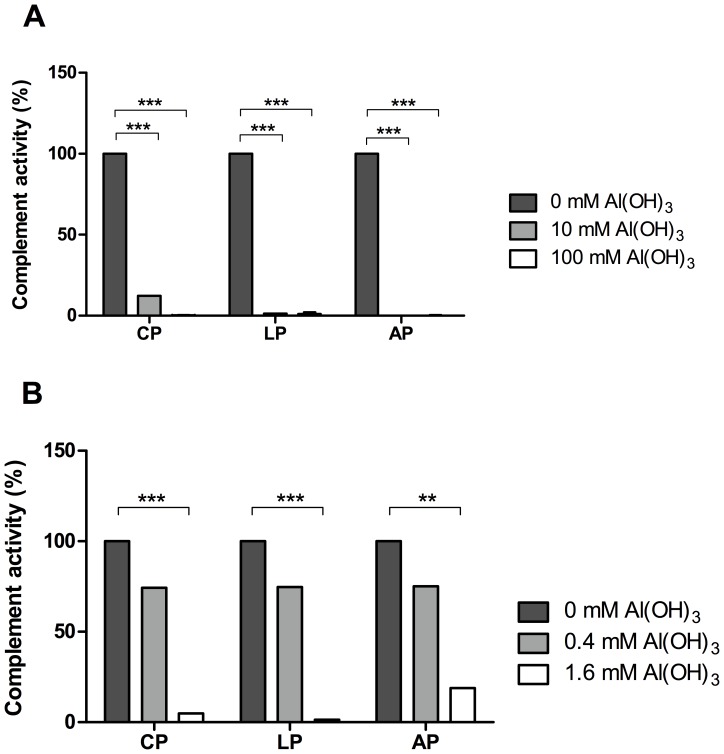
Al(OH)_3_ exhausts serum complement. The figure shows residual complement activity measured by MAC deposition by the three pathways (alternative pathway (AP), lectin pathway (LP), Classical pathway (CP)) in normal human serum (NHS) after incubation with different molarities of Al(OH)_3_ (37°C, 1 h, in milliQ water) and removal of the Al(OH)_3_-containing precipitate by centrifugation. 100% represents the complement activity without addition of Al(OH)_3_. A. Residual complement activity after incubation with vaccine-relevant amounts of Al(OH)_3_ (10 mM or 100 mM). The figure shows means ± SD of three independent experiments each with double determinations (the SDs are not graphically visible except for LP, 100 mM Al(OH)_3_). B. Residual complement activity after incubation with low amounts of Al(OH)_3_. The figure is one out of 3 experiments and shows the means of double determinations. Statistically significant differences were calculated using 2-way ANOVA and Bonferroni posttest (**p<0.01; ***p<0.001). Positive, negative and blank controls provided by the kit manufacturer were analysed in each experiment.

**Figure 3:**
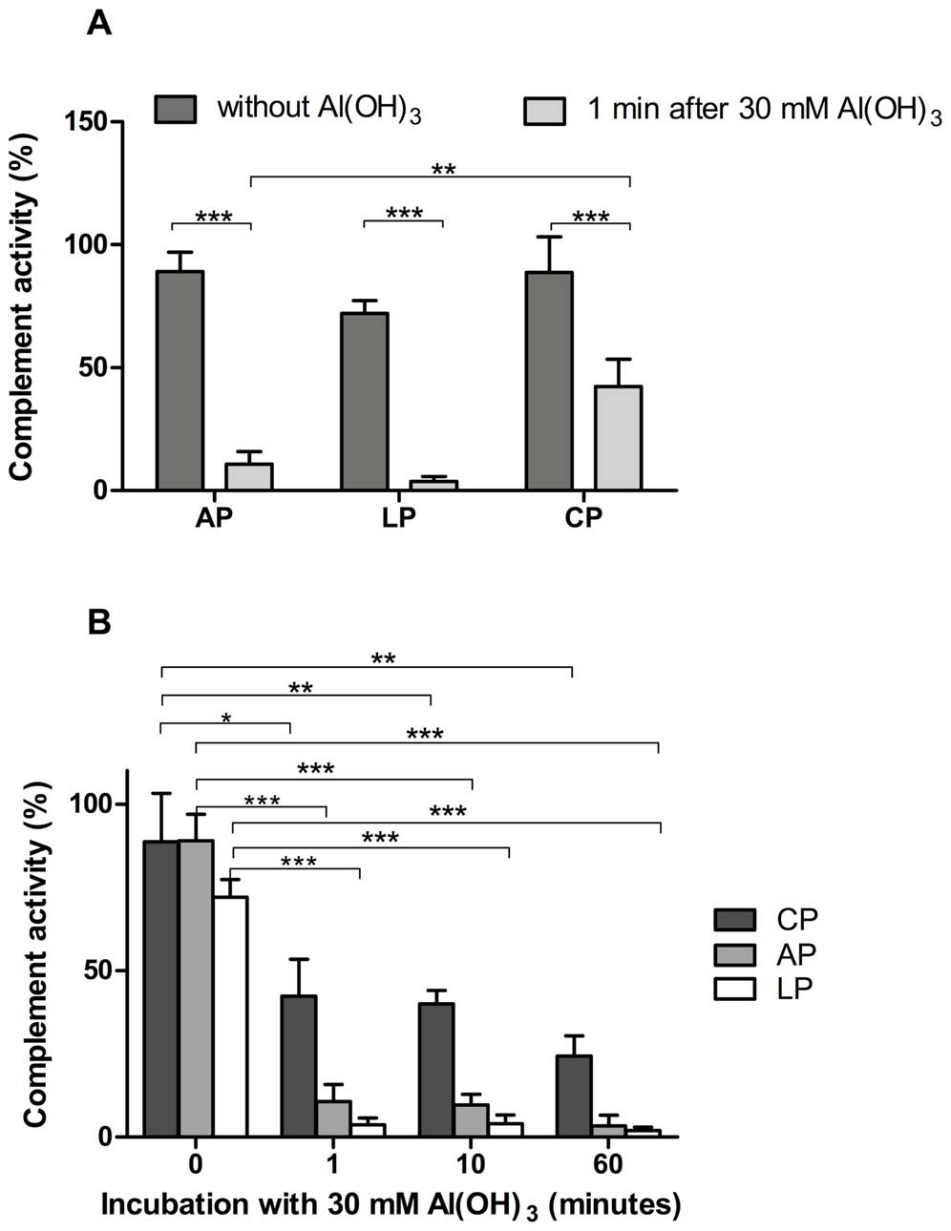
Time course for Al(OH)_3_ exhaustion of the three complement pathways. NHS was incubated with 30(OH)_3_ at 37°C for the indicated times and MAC deposition by the three pathways (alternative pathway (AP), lectin pathway (LP), classical pathway (CP)) was measured in the supernatant after centrifugation. A. Short term effect of Al(OH)_3_ on the three complement pathways. B. Long term effects of Al(OH)_3_ on complement activity. The figures show data from 3 independent experiments with means±SD. P-values for the reduction of complement activity were calculated using 2-way ANOVA and Bonferroni posttest (*p<0.05; **p<0.01; ***p<0.001). Positive, negative and blank controls provided by the kit manufacturer were analysed with each experiment.

In order to analyse the influence of Al(OH)_3_ on the complement pathways, different concentrations of Al(OH)_3_ were mixed with a complement-competent NHS for one hour at 37°C and the supernatants were analysed after the Al(OH)_3_-containing precipitates had been removed by centrifugation. The residual complement activity was determined individually for the three complement pathways by the ability to form the complement end-product MAC. Al(OH)_3_ was observed to reduce the ability of serum to deposit MAC in a concentration-dependent manner when added to serum diluted in the buffers optimal for the three pathways (1∶100 for CP and LP, 1∶18 for AP). We observed complete exhaustion of all pathways at 100 mM Al(OH)_3_ (Fig. 2A). At 10 mM Al(OH)_3_ the LP and AP were completely exhausted, while the CP retained some activity (Fig. 2A). At lower Al(OH)_3_ concentrations the residual activity of the three pathways varied more, due to the heterogenous nature of the reactions system (two-phase with amorphous Al(OH)_3_). However, it was reproducibly observed that the three pathways were significantly reduced already at 1.6 mM Al(OH)_3_ (Fig. 2B). In an attempt to distinguish between the three pathways we incubated serum with Al(OH)_3_ for different periods of time. Time course experiments at 37°C with 30 mM Al(OH)_3_ showed a rapid exhaustion of the AP and LP whereas the CP was exhausted more gradually (Fig. 3). Already after 1 min, the activities of the three pathways were reduced significantly and most for the AP and LP (Fig. 3A). The CP seemed to be exhausted more slowly, with some activity remaining even after 60 min (Fig. 3B). Similar results were obtained with lower concentrations of Al(OH)_3_ and at lower temperatures, except that the pathways were not completely exhausted (results not shown).

**Figure 4:**
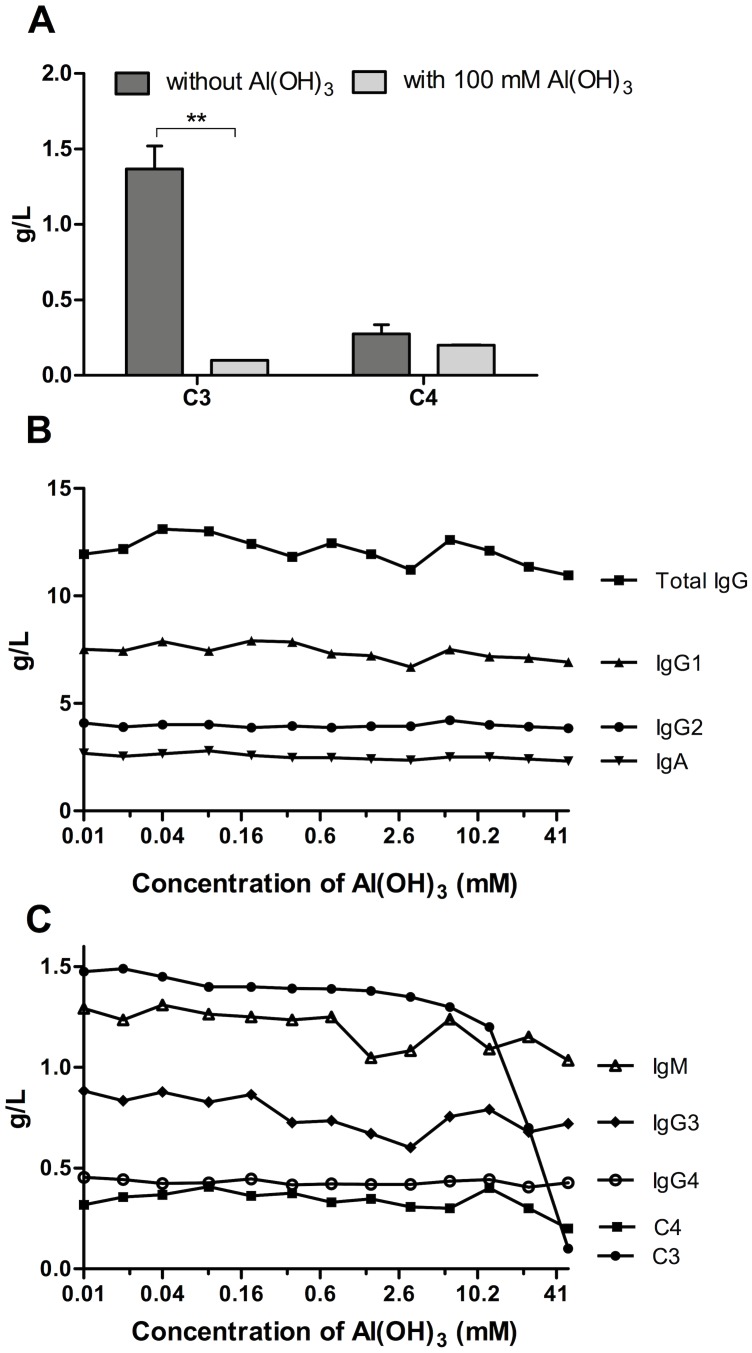
Al(OH)_3_ depletes C3 from serum. The concentrations of C3, C4 and different immunoglobulins were determined by nephelometry in NHS treated with Al(OH)_3_ at 37°C for one hour at the indicated molarities. The Al(OH)_3_-containing precipitate was removed by centrifugation prior to analysis of the supernatant. A. Concentration of C3 and C4 after treatment with 100 mM Al(OH)_3_. The figure shows the means ± SDs of two independent experiments. The statistical significance was calculated by 2-way ANOVA and Bonferrroni post-test (** p<0.01). B. Total IgG, IgG1, IgG2, IgA after Al(OH)_3_ treatment. C. IgM, IgG3, IgG4, C3 and C4 after Al(OH)_3_ treatment. B and C are from one out of two experiments.

Supernatants from Al(OH)_3_-treated serum were analysed and showed a reduced concentration of C3, depending on the Al(OH)_3_ concentration with only minor amounts of C3 remaining in serum treated with 100 mM Al(OH)_3_ for 1 hour (Fig. 4A), whereas no significant decrease in C4 was observed, suggesting that the AP is of major involvement. Al(OH)_3_ is known for its ability to adsorb proteins but the depletion of C3 did not occur merely by adsorption, as the levels of immunoglobulins, were not influenced by the Al(OH)_3_ treatment, even though, for example, the serum concentration, the isoelectric point and molecular weight of IgG_2_ are comparable to C3 (Fig. 4B,C).

**Figure 5:**
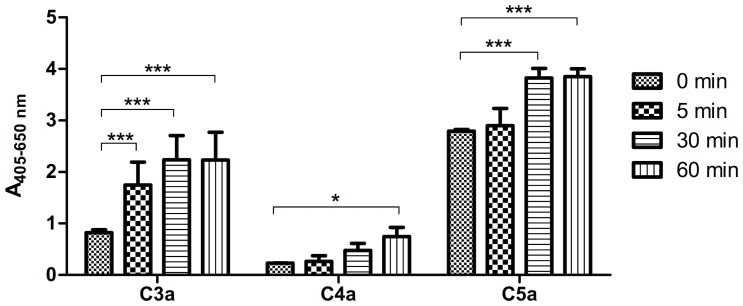
Time course of anaphylatoxin generation by Al(OH)_3_ in serum. The anaphylatoxins were measured in NHS after incubation with 30(OH)_3_ at 37°C for the indicated times and removal of the Al(OH)_3_-containing precipitate by centrifugation. High and low positive controls provided by the manufacturer and NHS without addition of Al(OH)_3_ were included as controls in all experiments. The figure shows means±SD of 2 independent experiments each with double determinations. P-values for the production of anaphylatoxins after 60 minutes were calculated using 2-way ANOVA and Bonferroni posttest (*p<0.05; ***p<0.001).

Complement activation was also demonstrated by the generation of anaphylatoxins. At 30 mM Al(OH)_3_ the generation of C3a and C5a showed a large increase with time, being evident already after 5 minutes (Fig. 5). C4a only showed a slight increase being significant only after 60 min. The formation of C3a and C5a increased with the Al(OH)_3_ concentration, whereas C4a showed no dependency on Al(OH)_3_ concentration (results not shown).

### Complement Components are Deposited on Al(OH)_3_ Precipitate

**Figure 6:**
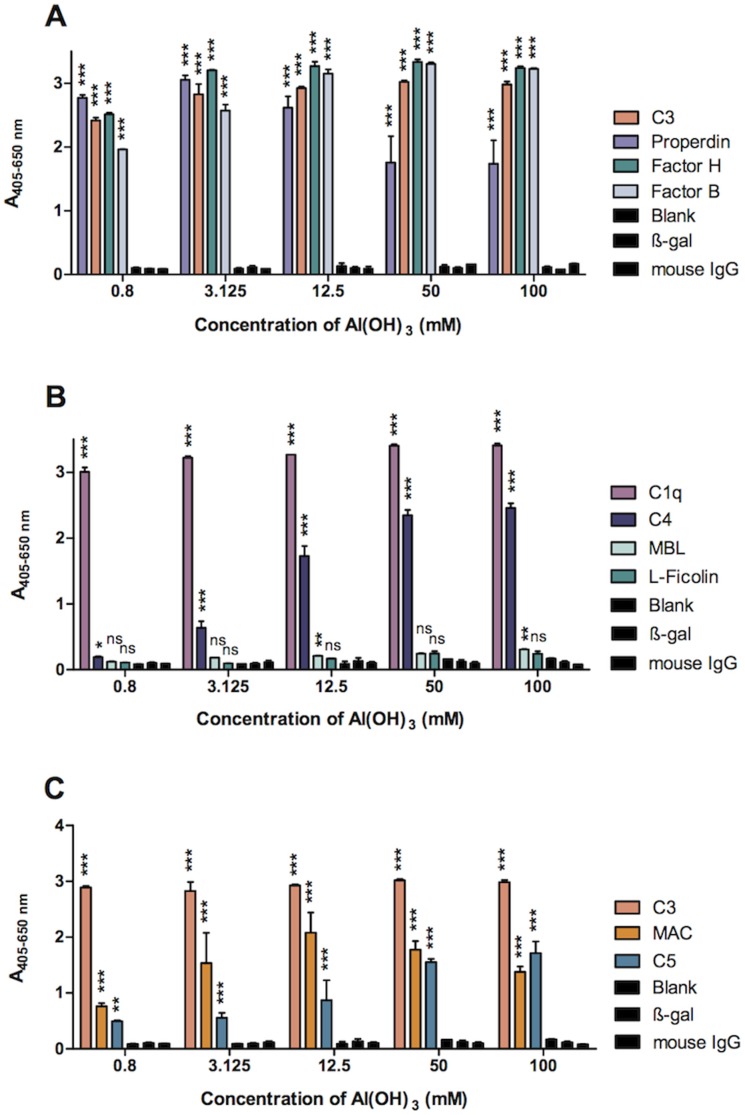
Complement components in serum are deposited on Al(OH)_3_. NHS was incubated with varying molarities of Al(OH)_3_ for 1 hour at 37°C, and the protein composition of the precipitates was analysed with monoclonal antibodies directed against the indicated complement components. Figure A shows results for AP components (C3, fB, fH, properdin). Figure B shows results for LP and CP components (mannan-binding lectin (MBL), L-ficolin, C1q, C4) and figure C shows results for C5 and MAC compared with C3. Data are presented as means±SD of 2 independent experiments each with double determinations. A blank control and two mock-antibody controls (mouse anti-β-galactosidase (β-gal) and total mouse IgG) were included in each experiment. 2-way ANOVA and Bonferroni posttest was used to assess the lowest Al(OH)_3_ concentration, in which the complement deposition was significant versus the control (β-gal) (*p<0.05; **p<0.01; ***p<0.001; ns = not significant).

**Figure 7:**
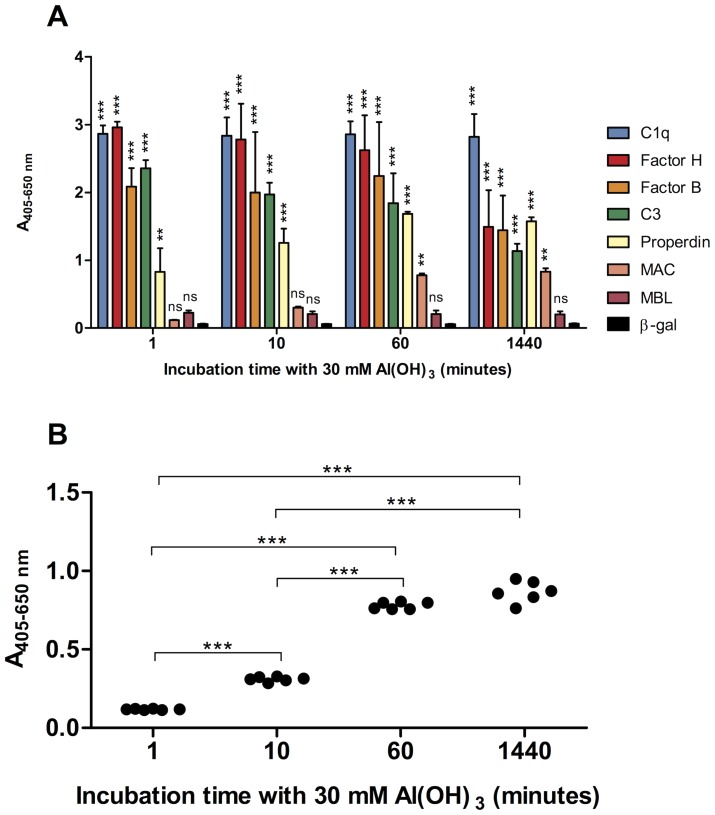
Time course for complement components deposition on Al(OH)_3_. A) Serum was incubated with 30 mM Al(OH)_3_ for the indicated times at 37°C and centrifuged. The protein composition of the precipitate was analysed using ELISA with monoclonal antibodies directed against the indicated complement components. Data are means±SD of 2 independent experiments. Controls were as in fig. 6. B) Time-dependent generation of the membrane attack complex (MAC The results are the means of 3 independent experiments. Statistical analysis was performed using 2-way ANOVA and Bonferroni posttest between the specific antibody versus control (β-gal) (A) or between time points (B) (**p<0.01; ***p<0.001; ns = not significant).

We investigated the complement components deposited on the amorphous Al(OH)_3_ precipitate after incubation with serum. The Al(OH)_3_-containing precipitates with deposited proteins were used directly for coating polystyrene microtitre plates and analysed for the presence of various complement components by ELISA (Fig. 6). AP components, C3 (or C3 cleavage products), factor B, properdin and factor H were found in major amounts at all concentrations of Al(OH)_3_ (Fig. 6A). LP recognition proteins, MBL and L-ficolin, were only detected at low levels, whereas C1q, the recognition molecule of the CP was detected at high levels (Fig. 6B). C4 was also deposited but increased gradually with increasing Al(OH)_3_ concentration (Fig. 6B). The downstream components of the complement cascade, C5 and MAC could also be demonstrated at significant levels (Fig. 6). Next, time experiments were carried out. C3, fB, fH, properdin and C1q showed rapid deposition on Al(OH)_3_ (Fig. 7A), whereas MAC deposited more slowly increasing significantly with incubation time (Fig. 7B). The increase in MAC deposition was found to be statistically significant from 1–10, 10–60 and 10–1440 minutes when analysed in 3 independent experiments (Fig. 7B).

**Figure 8:**
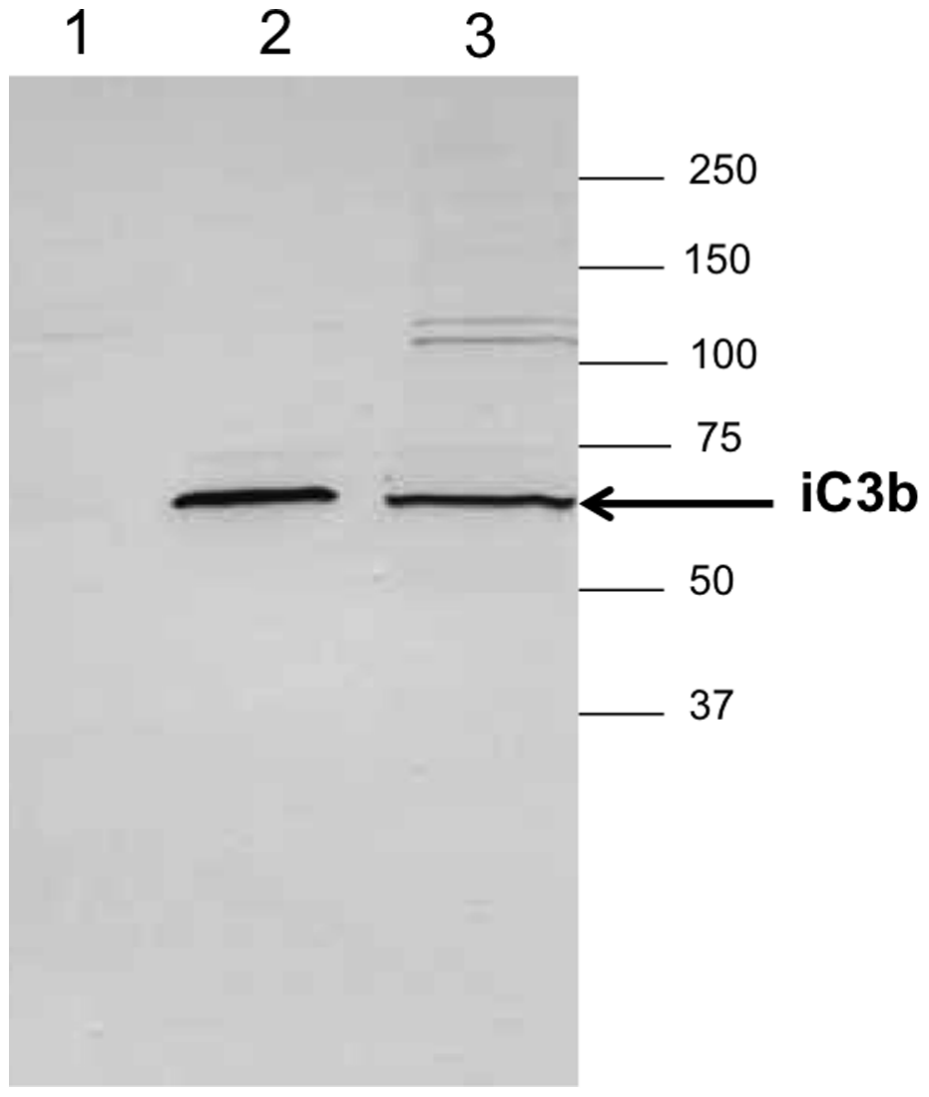
C3 cleavage products are present on the Al(OH)_3_ precipitate. The precipitate from serum incubated with 30(OH)_3_ was solubilised using EDTA and run on a reducing SDS-PAGE gel, blotted onto nitrocellulose and developed using a monoclonal antibody against C3d. First lane is a control sample with serum incubated with pH 5 buffer, second lane is authentic control iC3b and third lane is the solubilised sample from the Al(OH)_3_ precipitate.

The presence of complement components on the Al(OH)_3_ precipitate due to complement activation was confirmed using immuno-blotting with monoclonal antibodies directed against C3d, showing bands at ∼63 kDa (Fig. 8) indicative of iC3b generated from the C3 α-chain after C3 convertase and factor I cleavage. No bands of this Mw were observed in non-treated serum or serum treated with buffer of the same pH as alhydrogel (pH 5). The presence of C3 fragments on the Al(OH)_3_ was confirmed by mass spectrometry (results not shown).

### Complement Deposition by Al(OH)_3_ in Complement-depleted Sera

**Figure 9:**
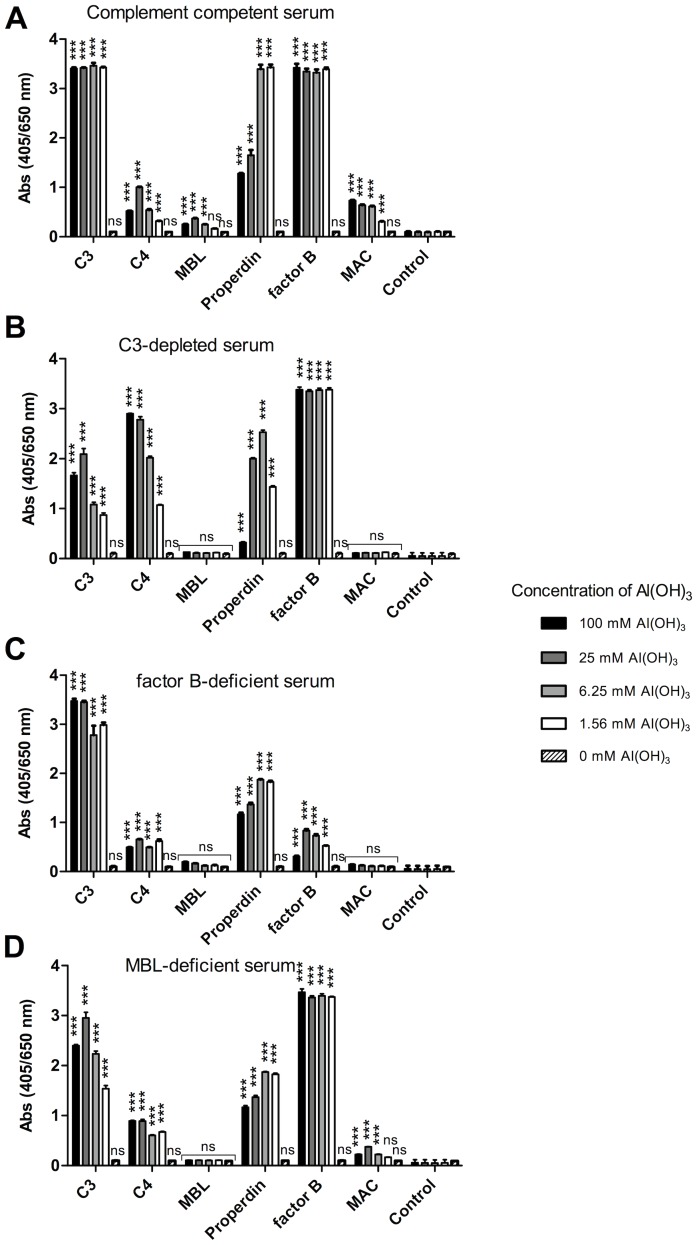
Al(OH)_3_-induced complement deposition in complement-compromised sera. Complement-competent NHS (A), a serum depleted for C3 (B), a factor B-depleted serum (C) and a mannan-binding lectin (MBL)-deficient serum (D) were treated with the indicated amounts of Al(OH)_3_ for 1 hour at 37°C and the Al(OH)_3_-containing precipitates were isolated by centrifugation and used for coating on a polystyrene surface and analysed for the complement components C3, C4, MBL, Properdin, factor B and membrane attack complex (MAC), using specific antibodies as previously described. The figure is one out of 2 experiments and shows the mean±SD of double determinations. 2-way ANOVA and Bonferroni posttest was used to assess the lowest Al(OH)_3_ concentration, in which the complement deposition was significant versus the control (β-gal) (***p<0.001; ns = not significant). Controls included were as described in the legend to fig. 6.

Complement-depleted sera (Table 1) were compared to the normal control serum with regard to complement activation induced by Al(OH)_3_. Compared to the control the C3-depleted serum (C3<0.05 mg/ml, CP activity ∼ 0% (Table 1)) still showed some immunoreactive C3 deposition when it was incubated with Al(OH)_3_ (Fig. 9B). This can be ascribed to remaining C3 degradation products and/or to the high sensitivity of the ELISA system used. Factor B deposition from the C3-depleted serum was comparable to the control, properdin deposition was decreased and C4 deposition was increased (Fig. 9B). A factor B-deficient serum showed very low factor B deposition, whereas properdin and C3 deposition were relatively high (Fig. 9C). The Al(OH)_3_ precipitate from an MBL-deficient serum showed a reduction in the MAC deposition and also a somewhat lower deposition of C3 and properdin, whereas factor B deposition was still high (Fig. 9D).

### The Effect of Inhibitors

**Figure 10:**
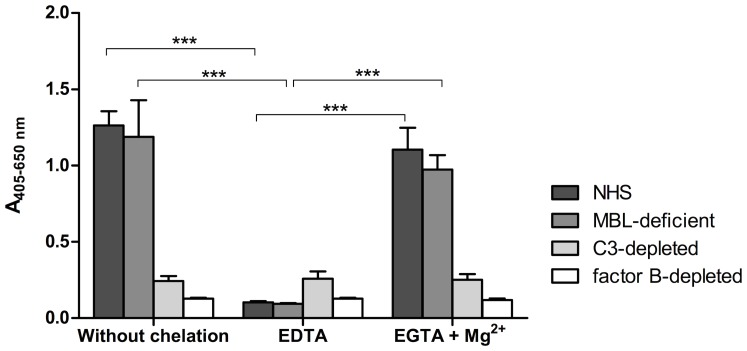
The effect of inhibitors on complement activation by Al(OH)_3_. Membrane attack complex (MAC) deposition was measured using a specific antibody on the precipitate of Al(OH)_3_-treated serum (30 mM Al(OH)_3_ for 1 hour at room temperature) with or without prior addition of 10 mM EDTA or 10 mM EGTA +10 mM MgCl_2_ comparing normal human serum with mannan binding lectin (MBL)-deficient serum, C3-depleted serum and fB-depleted serum. The figure shows means of 3 independent experiments±SDs. 2-way ANOVA and Bonferroni posttest showed a significant difference only for NHS and MBL-deficient serum after addition of EDTA (***p<0,001). Controls included untreated and lipopolysaccharide-treated serum.

Complement activation was also analysed in the presence of various inhibitors, both for NHS and complement compromised sera. When EDTA was added to NHS, to chelate divalent cations essential for complement cascade initiation and progression, the deposition of MAC on Al(OH)_3_ was significantly reduced (p = 0.0044)(Fig. 10). EGTA+Mg^2+^ is known to inhibit the C3 convertase of the classical and lectin pathways but allow alternative C3 convertase formation. Addition of EGTA+Mg^2+^ to NHS was found not to inhibit MAC deposition induced by Al(OH)_3_, compared to non-chelated serum (Fig. 10). An MBL-deficient serum showed reduced MAC deposition from Al(OH)_3_ in the presence of EDTA whereas MAC deposition was not influenced by EGTA+Mg^2+^ (Fig. 10). A commercial serum depleted for C3 showed less MAC deposition and no change in MAC deposition in the presence of EDTA or EGTA+Mg^2+^ (Fig. 10). Also, factor B-depleted serum showed no MAC deposition.

### Complement Deposition on Al(OH)_3_-containing Vaccines

**Figure 11:**
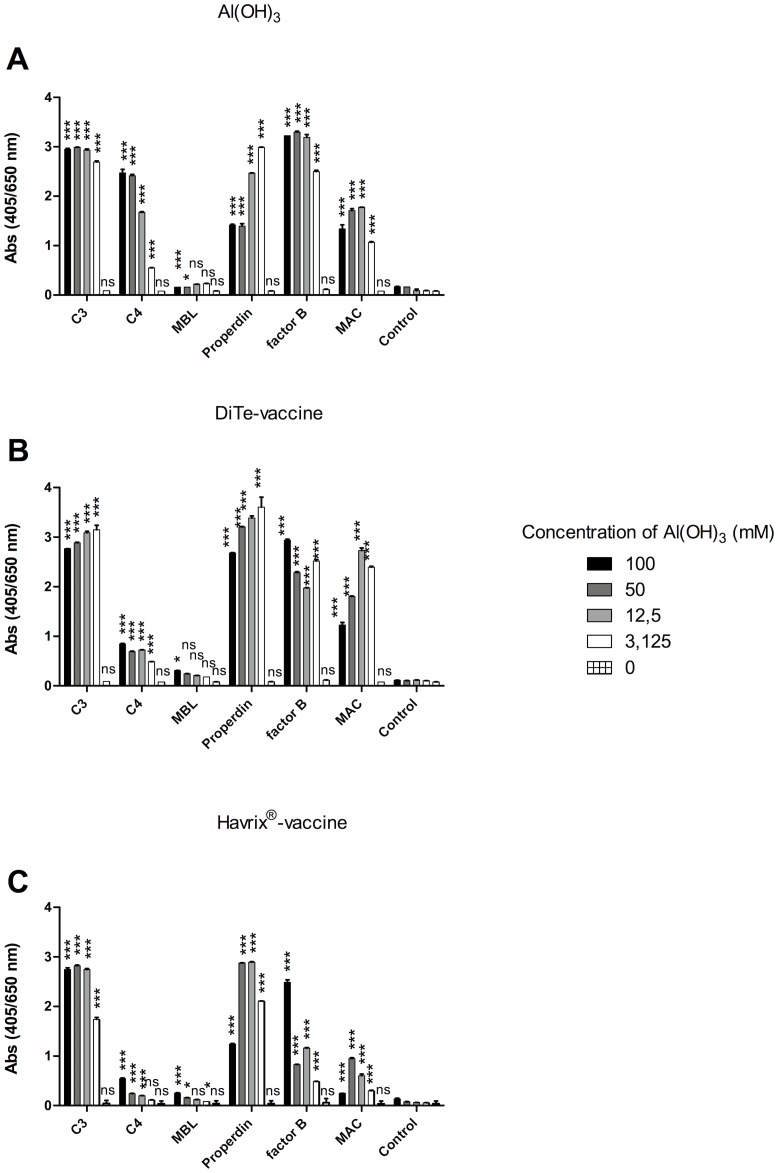
Deposition of complement occurs on human vaccines containing Al(OH)_3_. A human serum sample was treated with A) Al(OH)_3_, B) the Al(OH)_3_-containing vaccine diTe booster (diphtheria and tetanus vaccine) or C) the Al(OH)_3_-containing Havrix-vaccine. The Al(OH)_3_-containing precipitate was isolated by centrifugation and used for coating on a polystyrene surface and analysed for the complement components C3, C4, mannan-binding lectin (MBL), Properdin, factor B and membrane attack complex (MAC) using specific antibodies. The figure shows the mean of double determinations±SD from one of two independent experiments. Statistically significant differences were calculated using 2-way ANOVA and Bonferroni posttest for the specific antibody versus the control (β-gal) (*p<0.05, ***p<0.001).

To determine whether the ability of Al(OH)_3_ to exhaust serum complement could also be observed on Al(OH)_3_-containing vaccines for human use, NHS was mixed with Al(OH)_3_, tetanus toxoid/difteria toxoid adsorbed to aluminum hydroxide or Havrix vaccine and the precipitates were tested for complement deposition (Fig. 11). The vaccine precipitates showed essentially the same complement deposition characteristics as “naked” Al(OH)_3_ with minor variations due to different concentrations of Al(OH)_3_.

## Discussion

The complement system involves a large number of plasma proteins, which together combat pathogens and induce inflammatory responses. Three major pathways of complement cascades are known; the AP, the LP and the CP (Fig. 1). The AP is initiated by the presence of foreign surfaces (without complement inhibitors) leading to generation of the alternative C3-convertase. The LP and the CP are initiated when the recognition molecules MBL and C1q bind polysaccharides and immunoglobulins, respectively.

We show here that Al(OH)_3_ can activate the complement system when added to serum. Complete complement exhaustion was observed when measuring the residual complement activity after Al(OH)_3_ treatment. Al(OH)_3_ was found to exhaust complement components by complement activation as MAC was found to deposit time-dependently, suggesting that deposition on Al(OH)_3_ is not only a result of adsorption. Also, generation of anaphylatoxins clearly indicated complement activation together with deposition of C3 cleavage products on Al(OH)_3_. The ability of Al(OH)_3_ to activate the complement system in a serum sample was found to occur in a concentration- and time-dependent manner even at very low Al(OH)_3_ concentrations. The complement activation by Al(OH)_3_ was confirmed by analysing the Al(OH)_3_ precipitate showing detectable levels of many complement components and a MAC deposition increasing significantly with increasing incubation time. The precipitate of Al(OH)_3_-treated serum was also analysed by western blotting showing a distinct band of 63 kDa representing iC3b, which was not present in the untreated control serum sample. Complement activation was also analysed in the supernatant of Al(OH)_3_-treated serum, measuring the concentration of C3 and C5 in increasing concentrations of Al(OH)_3_ and a clear concentration-dependent reduction of C3 was observed. The immunoglobulin concentration in serum was not reduced, confirming that the reduction in C3 was not a result of C3 adsorption but of C3 consumption. The generation of anaphylatoxins was also investigated in supernatants from Al(OH)_3_-treated serum showing a clear induction of C3a and C5a with both increasing concentrations of Al(OH)_3_ and increasing incubation time. The complement activation assay suggested that the different pathways of the complement system might not be activated to the same degree by Al(OH)_3_. To investigate this further, Al(OH)_3_ activation of complement from serum was inhibited with EDTA (inhibits C3 convertase assembly) or EGTA+Mg^2+^ where only the AP can proceed. The MAC level on the Al(OH)_3_ precipitate was barely affected by the addition of EGTA+Mg^2+^, whereas no MAC was formed in the presence of EDTA, indicating a major involvement of the alternative pathway. This was confirmed by conducting the experiment with factor B-depleted serum, showing that no or very little MAC is deposited on Al(OH)_3_. The C4 level was measured in serum samples after Al(OH)_3_ treatment and removal of the precipitate and showed no distinct decrease in C4 concentration, suggesting little activation of the CP and LP. This was confirmed when the anaphylatoxin-levels were analysed and only small C4a increases could be detected. No or little increases in C4a concentration could be detected at increasing Al(OH)_3_ concentration or incubation-time. Collectively these data suggest a major involvement of the alternative pathway.

Despite the wide use of aluminum oxide-based adjuvants, their modes of action remain relatively poorly understood. When suspended in water, Al_2_O_3_ reacts with water to form aluminum hydroxide, Al(OH)_3_, which has the form of an amorphous precipitate. Aluminum hydroxide is thus the name for the chemical compound Al(OH)_3_, i.e. the hydrated form of aluminum oxide. Aluminum hydroxide cannot be isolated as a solid compound, it only exists as an amorphous precipitate, which appears to activate the complement system in a way similar to foreign surfaces. Complement activation by Al(OH)_3_ has been invoked by several groups [23]–[25] and in agreement with this, Chen et al. showed, that complement receptor-deficient mice had an impaired response to immunisation with antigen adsorbed to alum [29]. This is in agreement with reports showing that C3 and products of C3 play an important role in T_H_2 sensitization and antibody production [30], [31].

CP and LP activation by Al(OH)_3_ treatment has previously been suggested by Arvidsson and Ramanathan [24], [25]. Tengvall et al. did not observe properdin deposition on hydrated aluminum oxide but found, that C3 deposition was C1q-dependent [26]. These results are not necessarily in contradiction to our results, as all three pathways seem to be involved to some degree. However, they illustrate the difficulties in differentiating between the three pathways. In conclusion, we have confirmed that Al(OH)_3_ is able to exhaust serum complement and demonstrate a major importance of the AP. We propose that Al(OH)_3_ efficiently activates the AP and thus provide an explanation of how Al(OH)_3_ adjuvant can stimulate an immune response without being antigenic itself by providing a “surface” for complement activation, antigen opsonisation and stimulating antigen removal through complement receptors.
